# Maxillary denture flange and occlusal discrepancies of Vertex ThermoSens in comparison with conventional heat-cured denture base materials

**DOI:** 10.7555/JBR.32.20160132

**Published:** 2019

**Authors:** Hanadi A. Lamfon, Ibrahim M. Hamouda

**Affiliations:** 1. Maxillofacial and Oral Rehabilitation Department, Faculty of Dentistry, Umm Al-Qura University, Makkah Al Mukarramah 21955, Kingdom of Saudi Arabia; 2. Conservative Dentistry, Faculty of Dentistry, Umm Al-Qura University, Makkah Al Mukarramah 21955, Kingdom of Saudi Arabia; 3. Dental Biomaterials, Faculty of Dentistry, Mansoura University, Mansoura 35516, Egypt.

**Keywords:** denture distortion, flange discrepancy, acrylic resin, occlusal discrepancies, denture base

## Abstract

This study was conducted to investigate the maxillary denture bases and occlusal discrepancies using the Vertex Thermosens in comparison with the conventional polymethyl-methacrylate materials. Twenty maxillary denture bases were prepared from the Vertex ThermoSens and a conventional heat-cured denture base materials. Acrylic maxillary second molars were arranged in their respective positions on the ridge. After curing of both types of denture bases, they were deflasked with their respective master casts. Reference points were prepared for measurements of the antero-posterior and cross-arch dimensions at the denture borders using caliper device. Furthermore, the teeth discrepancies were measured between reference points in the ligual aspect of the second maxillary molars. The recorded data was analyzed using SPSS statistical software version 20. The results showed initial shrinkage of both denture bases in the antero-posterior and cross-arch dimensions immediately after decasting. This contraction was compensated gradually during storage in water up to 2 weeks. Regarding the variable time, there was a significant difference between the tested materials. Moreover, the results revealed occlusal discrepancies and shifting of teeth inward immediately after decasting, followed by outward movement after storage in water for 2 weeks. Regarding the variables time and materials, there were significant differences. Both materials exhibited inward shrinkage in the antero-posterior and cross-arch dimensions immediately after decasting. Both denture bases showed inward shifting of teeth immediately after decasting, followed by outward movement after storage in water up to 2 weeks.

## Introduction

Heat-cured and self-cured acrylic resins have been the common materials for denture base construction. Unfortunately, all resins used in dentistry undergo shrinkage during processing. Poor fitted dentures were evident as a result of shrinkage^[1]^.

The major objective for construction of complete dentures is to obtain a denture base that conforms the supporting tissues to a high degree of accuracy^[2]^. The properties of the denture bases are generally more important to the performance of the denture as it controls retention, mechanical properties, and biocompatibility^[3]^. It is believed by many authors that intimate tissue contact and peripheral seal of the denture base comprise the most critical retentive factors^[2]^.

Complete dentures are affected by the dimensional changes of the acrylic resins. These changes occur during or after processing. Volumetric shrinkage and the linear shrinkage are the main factors affecting denture stability. Linear shrinkage exerts significant effects on denture base adaptation and cuspal interdigitation^[1]^.

The magnitude of linear shrinkage is determined by measuring the distance between two predetermined reference points in the second molar regions of a complete tooth arrangement. Following polymerization of the denture base resin and removal of the prosthesis from the master cast, the distance between these reference points is measured once again. The difference between pre- and postpolymerization measurements is recorded as linear shrinkage^[1]^.

There are several modifications of denture base materials, including the conventional acrylic resins, high impact resins, glass fibers-reinforced resins and metallic-reinforced resins. Recent advances of poly-methyl methacrylate had been used for denture base construction. “ProBase Hot” was introduced as a new denture base material which is supposed to set up a higher standard of quality for the processing properties, accuracy of fitness, and stability of shape than heat cured denture base materials^[4]^. Finally, the latest form of denture base materials is the Vertex^TM^ ThermoSens thermoplastic material.

There was a little information in the literatures about the Vertex ThermoSens denture base materials. The null hypothesis of this study was that Vertex ThermoSens material is superior in maxillary denture flange adaptation and occlusal stability to the conventional denture base material. Therefore, the aim of this study was to investigate the discrepancies of the maxillary denture base and occlusal plane of dentures made from the newly introduced Vertex ThermoSens in comparison with the conventional polymethyl-methacrylate denture base materials.

## Materials and methods

Twenty identical maxillary stone casts were produced from a standard metallic mold. The maxillary residual ridge was free of any obvious ridge undercuts or surface irregularities and had a smooth U-shaped, well-formed arch. The master casts were divided into two groups, 10 casts each. The first group was assigned for construction of 10 denture bases from the conventional heat-cured acrylic resin as a control group (compression molding technique). The second group was assigned for construction of 10 denture bases from the newly introduced Vertex ThermoSense denture base material (injection molding technique).

Baseplate wax was constructed on one master cast using one sheet of wax 2 mm thick (Tru Wax, Dentsply International Inc., York, PA, USA). Anatomic acrylic maxillary second molars were arranged in their respective positions on the ridge (Dentsply International Inc., York, Pa.). The wax base thickness was preserved as 1.25 mm. Small amounts of wax were added to fix the teeth in their respective positions^[5]^. Two wax sprues with 10 mm diameter were attached on the back of tuberosities. The system (model, base plate wax and teeth) was duplicated using polyvinyl siloxane (Silastic E; Dow Corning, Midland, Mich, USA) to prepare 20 identical denture base wax. After silicon curing, waxed denture base and model has been removed. This silicon was used as a standard template for next waxed denture base specimens. The teeth of the same sizes were arranged in their respective positions in the silicone mold. This silicone mold was placed on 20 stone casts where the teeth and model were fitted in location. Molten base plate wax was poured through the sprues and after cooling the wax replicas of denture were obtained.

The waxed denture bases were invested with dental plaster and dental flasks using compression molding technique. Stone cap were prepared over the cusps of the maxillary teeth. Waxed denture bases were washed up in boiling water for 10 minutes. The flasks were opened and wax was eliminated. After removing the wax with boiling water and separating medium application, the first group was constructed from heat-polymerized resin, and Major.base 20 (Major Prodotti Dentari S.p.A. Italy) were packed according to the manufacturer^,^ s instructions. The powder/liquid was mixed at a ratio (3:1) andpacked, and the flasks were dipped in boiling water at 100 °C for 30 minutes. The flasks were cooled slowly and the denture bases were deflasked with their respective casts in position. While the denture bases remained in their position, the casts were trimmed from the cast-base to expose the border of the denture flanges.

The second group was constructed from Vertex ThermoSens (Vertex^TM^ ThermoSens, Vertex-Dental, Netherlands) according to the manufacturer^,^ s instructions. This system used special metallic flasks with posterior wax sprue for injection of the material inside the plaster molds. Vertex^TM^ ThermoSens is based on injection technique, with an automatic or manual injection machine. The model and flasks were prepared according to the standard procedures of the dental technique. Since there is no chemical bonding between synthetic acrylic teeth and Vertex™ ThermoSens, a mechanical bonding must be obtained. For injection of the Vertex™ ThermoSens into the flask, wax sprue should be used. The main sprue was about 9.5 mm and side sprues was 4.5 mm^[6]^. The material was heated at 270 °C–280 °C within 18 minutes and injected automatically at a pressure of 8.5 bar. The flasks were cooled slowly and the dentures were deflasked with their respective casts.

**Fig.1:**
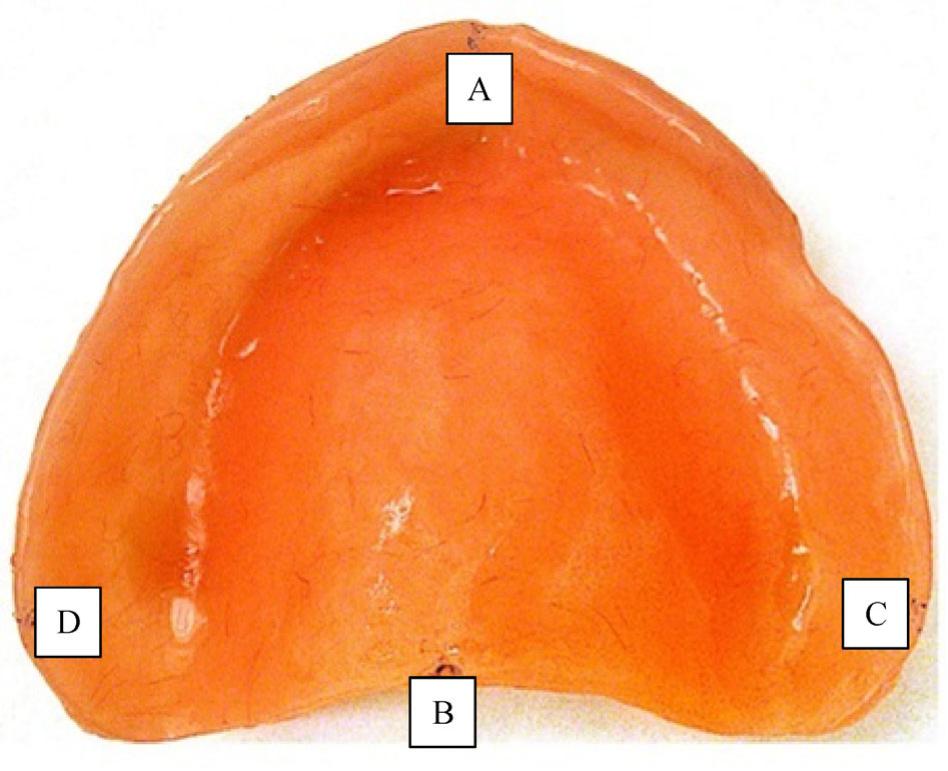
The antero-posterior reference points (A-B) and the right to left (C-D, flange-flange) reference points

The models were trimmed to expose the denture borders at the regions of the tuberosities, labial frenum notch and the postdam. To assess the anterior-posterior dimensional changes, two reference points were prepared in the midline at the fitting surface of the bases. The first point located at the labial frenum notch (point A) and the second point in midline of the postdam (point B). Also, two reference points (C and D) were prepared in the internal surface of the buccal pouch part of the border of the denture flange (***Fig. 1***)^[6]^.

The lengths between A, B, C and D points were standardized in all dentures by using metallic bar. The distances between these points in the fitting surface were measured by dial caliper (Mitutoy, Us Ms00 13, 500 series, Japan). The caliper has two jaws where one is fixed and the other is movable. The sliding jaw was moved by pressing the thumb on the bump on the bottom. The caliper was used for reading of the distance between centers of these points in antro-posterior direction (A and B) and right-left direction (C and D) and the results were recorded. The unit of measurement was the millimeter with precision of 0.01. The distances were measured before deflasking, while the dentures were still on their models, after removal of the denture from their casts, and 1 week and 2 weeks of water storage after decasting.

**Fig.2:**
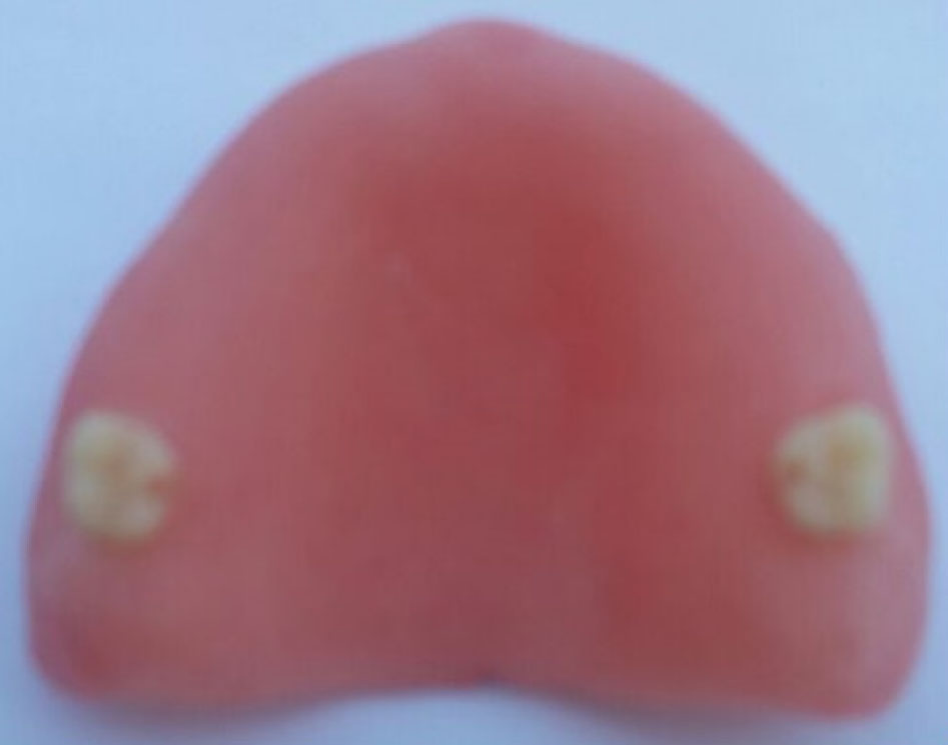
Reference points for molar-molar measurements

Immediately after the stone casts were deflasked with the dentures still in place, reference notches were prepared in the lingual aspect of the most distal molar tooth on either side of the arch (***Fig. 2***). With a caliper device, the molar-molar (M-M) cross arch linear distance was measured. The dentures were removed from their casts , the M-M cross arch linear distance was again measured^[5]^. Measurements were done after storage in water for 1 and 2 weeks.

### Statistical analysis

The recorded data were analyzed using 2-way ANOVA and LSD tests to detect the significant differences between the two tested materials at the different periods.

## Results

The results of the antero-posterior dimensions (A-B) are presented in ***Table 1***. The statistical analysis of the results using 2-way ANOVA showed a significant difference between the conventional heat-cured acrylic resin and the Vertex ThermoSens regarding the antero-posterior dimensions (*P*≤0.001). Both denture base materials showed significant shrinkage in the antero-posterior (A-B) dimensions after decasting (*P*≤0.05). Both denture bases exhibited gradual expansion in the antro-posterior dimension during the 2 weeks of water immersion. There were no significant differences between the tested materials in the antro-posterior dimension (*P*>0.05). Regarding the time factor, there were significant differences between the different testing periods in the antro-posterior dimension (*P*≤0.05).

**Tab.1:** The antero-posterior dimensions (mm) at different periods

Materials	Before decasting	After decasting	1 week storage in water	2 week storage in water	*F*-value	*P*-value
Major. base 20	42.46±0.60^a^	42.17±0.50^b^	42.38±0.50^a^	42.62±0.60^a^	454,316.5	*P*≤0.001
Vertex ThermoSens	42.48±0.40^a^	42.12±0.80^b^	42.36±0.50^a^	42.54±0.60^a^		

The mean difference is significant at the 0.05 level. The means with different superscripted letters are significantly different.

The results of the cross arch dimensions (flange-flange, C-D) are presented in ***Table 2***. The statistical analysis of the results using 2-way ANOVA showed a significant difference between the conventional heat-cured acrylic resin and the Vertex ThermoSens regarding the cross arch dimensions (*P*≤0.001). Both denture base materials showed significant shrinkage in cross arch dimensions (flange-flange, C-D) after decasting (*P*≤0.05). Both denture bases exhibited gradual expansion in the cross arch dimensions (flange-flange, C-D) during the 2 weeks of water immersion. There were no significant differences between the tested materials in the cross arch dimensions (flange-flange, C-D) (*P*>0.05). Regarding the time factor, there were significant differences between the different testing periods in the flange-flange dimension (*P*≤0.05).

**Tab.2:** Flange-flange distance (mm) at different periods

Materials	Before decasting	After decasting	1 week storage in water	2 week storage in water	*F*-value	*P*-value
Major. base 20	61.76±0.70^a^	61.62±0.90^b^	61.66±0.80^b^	61.68±0.70^a^	199,615.9	*P*≤0.001
Vertex ThermoSens	61.75±0.80^a^	61.60±0.80^b^	61.63±0.70^b^	61.78±0.80^a^		

The mean difference is significant at the 0.05 level. The means with different superscripted letters are significantly different.

The results of the inter-occlusal (molar-molar) dimensions are presented in ***Table 3***. The statistical analysis of the results using 2-way ANOVA showed a significant difference between the conventional heat-cured acrylic resin and the Vertex ThermoSens in the molar-molar dimensions (*P*≤0.001). Both denture base materials showed significant occlusal discrepancies after decasting (*P*≤0.05). Both denture bases exhibited gradual expansion in the molar-molar dimensions during the 2 weeks of water immersion. There were significant differences between the tested materials in the teeth discrepancy all over the tested materials (*P*≤0.05). Regarding the time factor, there were significant differences between the different testing periods in the teeth discrepancy (*P*≤0.001). The interaction of the time and materials variables was significantly different in this test (*P*≤0.001).

**Tab.3:** Molar-molar distance (mm) at different periods

Materials	Before decasting	After decasting	1 week storage in water	2 week storage in water	*F*-value	*P*-value
Major. base 20	42.40±0.54^b,e^	41.21±0.54^f^	42.32±0.53^b^	42.38±0.53^b^	5.72	*P*≤0.001
Vertex ThermoSens	42.08±0.65^d^	41.38±0.61^c^	42.39±0.54^b^	42.59±0.52^a^		

The mean difference is significant at the 0.05 level. The means with different superscripted letters are significantly different. The superscripted letter a assigned for the highest mean followed by b, c, d, e, and f for the lowest mean.

## Discussion

Vertex™ ThermoSens is a thermoplastic material to be used for dental prosthesis. The product is based on a compounded mixture of polyamide and pigments, and will be used as a thermoplastic in the injection technique. Since the product does not contain residual monomer, the product is suitable for people allergic for residual monomer. Vertex™ ThermoSens is intended for removable full and partial dentures as well as splints, telescope constructions and temporary crown and bridge constructions.

The pigments and the vines in Vertex™ ThermoSens rigid are built in the raw material through the production process, which leads to an equal distribution of pigments and vines in the denture^[7]^. The use of calipers for measurements was proved to be a highly reliable instrument to assess oral vertical dimension^[8]^.

Better fitness of the dentures in the patient mouth depends on the adaptation of the denture base to their casts^[9]^. The processing changes and denture base distortion that occur when the polymerized dentures are removed from the casts are considered the major disadvantages of acrylic resin, and these factors can also causes teeth displacement from its position^[10]^. Teeth showed significant movement during processing of acrylic resin dentures. Overall, the movement of teeth in shallow palatal form dentures was in palatal direction, whereas in deep palatal form dentures, the movement of teeth was in buccal direction^[11]^. The dimensional change that occurs during the denture processing is a critical factor in the retention and stability of the complete denture^[12]^.

When methyl methacrylate monomer is polymerized to form polymethyl methacrylate, the density of the mass changes from 0.94 to 1.19 g/cm^3^. This change in density results in a volumetric shrinkage of 21%^[1]^. The differential in thermal contraction coefficient between the gypsum mold and the acrylic resin is believed to be the cause of residual stresses accumulated within the processed dentures. Releasing of these stresses when the denture is separated from the cast considered to be the main cause of denture warpage^[1^,^13]^. The larger amounts of stresses may be generated at the marginal borders rather than at the ridge crests leading to larger strains after denture deflasking^[14]^. There was an interaction between the anterior and posterior regions of the denture. The posterior region showed greater discrepancy when compared to the anterior region. Regardless of the technique and resin used for denture base construction, the greater discrepancy was observed in the posterior region of the palate^[15]^.

In addition to volumetric shrinkage, one also must consider the effects of linear shrinkage. Linear shrinkage exerts significant effects on denture base adaptation and cuspal interdigitation^[1^,^3]^. The greater the linear shrinkage, the greater is the discrepancy observed in the initial fit of a denture. Examination of the polymerization process indicates thermal shrinkage of resin is primarily responsible for the linear changes observed in heat-activated systems. During the initial stages of the cooling process, the resin remains relatively soft. Consequently, the resin mass contracts at about the same rate as the surrounding dental stone^[1^,^3^–^4]^. Following polymerization of the denture base resin and removal of the prosthesis from the master cast, the distance between these reference points are measured once again. The difference between pre- and postpolymerization measurements is recorded as linear shrinkage. The greater the linear shrinkage, the greater is the discrepancy observed in the initial fit of a denture^[1^,^5]^.

The results of this study indicated contraction in the antero-posterior and cross arch lateral measurements after removal of the denture bases from their casts. This may be explained on the basis of the linear shrinkage produced from the thermal shrinkage of the denture base materials during cooling^[1^,^3]^. This linear shrinkage will be recovered during storage of the denture bases in water up to two weeks^[3^,^16]^. Thermoplastic acrylic resins used as materials for non-metal clasp dentures are applicable in the oral cavity environment because of low water sorption rate which is similar to those of the thermoplastic polyamide and conventional heat-polymerized acrylic resins^[16]^. After 2 weeks of water storage the antero-posterior and cross-arch lateral measurement became comparable to the measurements before decasting.

Tooth movements of processed acrylic resin dentures were significantly affected by the palatal form. After deflasking, in shallow palatal form, teeth moved palatally or toward each other, whereas in wide palatal form, the teeth showed a buccal or outward displacement. After finishing and polishing, teeth had a tendency to move buccally regardless of the palatal form. The overall movement of teeth during the processing of dentures was in palatal direction with shallow palatal form and in buccal direction with deep palatal form^[11]^. The results of this study indicated that, the teeth were moved palatally after decasting and subsequently moved buccally during storage in water up to 2 weeks. Most of occlusal discrepancies were relieved with water storage.

Dimensional change of the denture base was influenced by the storage period in water^[17]^. Occlusal discrepancies are produced in complete dentures as a result of processing procedures. The processing warpage and denture base distortion that occur when the polymerized dentures are removed from the cast are the major disadvantages of acrylic resin. These factors can modify the teeth position. Careful processing can minimize changes in planned occlusal contacts of the opposing artificial posterior teeth^[18]^.

Polymethyl methacrylate exhibits a water sorption value that produces significant effects in polymerized resins. It has been estimated that for each 1% increase in weight produced by water absorption, acrylic resin exhibits a linear expansion of 0.23%. Linear expansion caused by water absorption is approximately equal to the thermal shrinkage encountered as a result of the polymerization process^[1]^. The clinical relevance of this result can be significant for the comfort of patients, considering that the main purpose of this study was to identify the process that improves the stability of the tooth in the complete dentures.

In conclusions, within the limits of this study, the following conclusions could be noted: (1) The null hypothesis of this study was rejected. (2) Both denture base materials exhibited inward shrinkage in the antero-posterior and cross-arch dimensions immediately after removal from their master casts. (3) Both denture base materials showed gradual outward expansion after storage in water for 2 weeks to compensate the initial contraction. (4) Both denture bases showed inward shifting of teeth immediately after decasting, followed by outward movement after storage in water up to 2 weeks.
